# PREOPERATIVE CHEMOTHERAPY VERSUS UPFRONT SURGERY FOR ADVANCED GASTRIC CANCER: A PROPENSITY SCORE MATCHING ANALYSIS

**DOI:** 10.1590/0102-672020230018e1736

**Published:** 2023-07-07

**Authors:** Stefany Hong, Marina Alessandra Pereira, Carolina Ribeiro Victor, João Vitor Antunes Gregório, Bruno Zilberstein, Ulysses Ribeiro, Luiz Augusto Carneiro D'albuquerque, Marcus Fernando Kodama Pertille Ramos

**Affiliations:** 1 Universidade de São Paulo, Faculty of Medicine, Hospital das Clínicas, Department of Gastroenterology – São Paulo (SP), Brazil; 2 Universidade de São Paulo, Faculty of Medicine, Hospital das Clínicas, Department of Radiology and Oncology – São Paulo (SP), Brazil.

**Keywords:** Stomach neoplasms, Surgical procedures, operative, Propensity score, Neoadjuvant therapy, Drug therapy, combination, Neoplasias gástricas, Procedimentos cirúrgicos operatórios, Pontuação de propensão, Terapia neoadjuvante, Quimioterapia Combinada

## Abstract

**BACKGROUND:**

Surgical resection remains the main curative therapeutic modality for advanced gastric cancer. Recently, the association of preoperative chemotherapy has allowed the improvement of results without increasing surgical complications.

**AIMS:**

To evaluate the surgical and oncological outcomes of preoperative chemotherapy in a real-world setting.

**METHODS:**

A retrospective review of gastric cancer patients who underwent gastrectomy was performed. Patients were divided into two groups for analysis: upfront surgery and preoperative chemotherapy. The propensity score matching analysis, including 9 variables, was applied to adjust for potential confounding factors.

**RESULTS:**

Of the 536 patients included, 112 (20.9%) were referred for preoperative chemotherapy. Before the propensity score matching analysis, the groups were different in terms of age, hemoglobin level, node metastasis at clinical stage- status, and extent of gastrectomy. After the analysis, 112 patients were stratified for each group. Both were similar for all variables assigned in the score. Patients in the preoperative chemotherapy group had less advanced postoperative p staging (p=0.010), postoperative n staging (p<0.001), and pTNM stage (p<0.001). Postoperative complications, 30- and 90-days mortality were similar between both groups. Before the propensity score matching analysis, there was no difference in survival between the groups. After the analysis, patients in the preoperative chemotherapy group had better overall survival compared to upfront surgery group (p=0.012). Multivariate analyses demonstrated that American Society of Anesthesiologists III/IV category and the presence of lymph node metastasis were factors significantly associated with worse overall survival.

**CONCLUSIONS:**

Preoperative chemotherapy was associated with increased survival in gastric cancer. There was no difference in the postoperative complication rate and mortality compared to upfront surgery.

## RESUMO

**RACIONAL:**

A ressecção cirúrgica continua sendo a principal modalidade terapêutica curativa para o câncer gástrico avançado. Recentemente, a associação de quimioterapia pré-operatória tem permitido a melhora dos resultados sem aumentar as complicações cirúrgicas.

**OBJETIVOS:**

Avaliar os resultados cirúrgicos e oncológicos da quimioterapia pré-operatória em um cenário do mundo real.

**MÉTODOS:**

Realizou-se uma revisão retrospectiva de pacientes com câncer gástrico submetidos à gastrectomia. Os pacientes foram divididos em dois grupos para análise: cirurgia inicial e quimioterapia pré-operatória. A análise por escore de propensão, incluindo 9 variáveis, foi aplicada para ajustar possíveis fatores de confusão.

**RESULTADOS:**

Dos 536 pacientes incluídos, 112 (20,9%) foram encaminhados para quimioterapia pré-operatória. Antes da análise por escore de propensão, os grupos eram diferentes em termos de idade, nível de hemoglobina, *status de node metastasis at clinical stage* e extensão da gastrectomia. Após a análise, 112 pacientes foram estratificados para cada grupo. Ambos foram semelhantes para todas as variáveis atribuídas no escore. O grupo da quimioterapia pré-operatória apresentou estágios *postoperative p staging* (p=0,010), *postoperative n staging* (p<0,001) e pTNM menos avançados (p<0,001). As complicações pós-operatórias e a mortalidade em 30 e 90 dias foram semelhantes entre os grupos. Antes da análise por escore de propensão, não houve diferença na sobrevida entre os dois grupos. Após a análise, o grupo da quimioterapia pré-operatória apresentou melhor sobrevida global em comparação ao grupo da cirurgia inicial (p=0,012). As análises multivariadas demostraram que a categoria American Society of Anesthesiologists III/IV e a metástase linfonodal foram fatores significativamente associados à pior sobrevida global.

**CONCLUSÕES:**

A quimioterapia pré-operatória foi associada à maior sobrevida no câncer gástrico. Não houve diferença na taxa de complicações pós-operatórias e mortalidade em comparação com a cirurgia inicial.

## INTRODUCTION

Gastric cancer (GC) is the fifth most common cancer and the fourth cancer-related death worldwide^30^. Surgical treatment persists as the main curative treatment modality, but in the last two decades, the association with systemic chemotherapy (CMT) has been adopted as the standard treatment for patients with advanced GC and lymph node (LN) metastasis^7,23^.

Among the modalities of CMT, preoperative CMT, including neoadjuvant or perioperative regimens, has been recommended instead of upfront surgery^3,15^. This approach involves the possibility of tumor downstaging, an increase in the R0 resection rate, and achieving a better rate of adherence to CMT with completion of the proposed treatment^2,23,28^. Furthermore, preoperative CMT can lead to a significant increase in survival, without impairing surgical results, postoperative morbidity, and mortality rates^5^.

Despite the several evidence in favor of the preoperative CMT approach, the main randomized clinical trials (RCT) show great heterogeneity that can limit comparisons between studies and generalization of results^3,15,29,33^. Differences regarding the type of drugs and CMT schemes, the extent of lymphadenectomy, and the inclusion of distal esophagus and gastroesophageal junction (GEJ) tumors cases are some of the sources of bias. Also, most GC trials related to surgical treatment are conducted in Asian countries, in large, specialized centers that have extensive expertise in surgery and lymphadenectomy with good survival rates even without preoperative CMT. In turn, trials about preoperative CMT are mainly conducted in Europe, raising questions about biological behavior and the feasibility of adequate lymphadenectomy in different ethnic populations^10^.

Another aspect also addressed about preoperative CMT concerns the rate of postoperative complications. Although most of the RCTs show no difference in morbidity and mortality compared to upfront surgery, it is known that most patients included in clinical trials do not correspond to patients with GC treated in our real-world setting, since they tend to have ideal clinical characteristics^19^. Therefore, potential selection bias in such studies has not been approached.

In our service, more than 80% of patients are diagnosed with advanced GC, and some of them have consumptive syndrome and high nutritional risk. In this scenario, lymphadenectomy has been adopted since the 1980s following the precepts of the Japanese Gastric Cancer Association. At the same time, improvements in nutritional support, perioperative care, and multidisciplinary team training were implemented. Recently, we reported the results of our service, where the benefit in survival achieved with adequate lymphadenectomy was confirmed, with morbidity and mortality rates comparable to other western centers^27^. Therefore, the adoption of preoperative CMT in our service was done with caution and presented as a new challenge^13,16^. Thus, this study aimed to evaluate the short- and long-term outcomes of preoperative CMT for locally advanced GC compared to upfront surgery, using propensity score matching (PSM) analysis to adjust for potential confounding factors.

## METHODS

A retrospective review of all GC patients who underwent surgical treatment from 2009 to 2021 was performed. The source of information was the prospectively maintained hospital research database. Only histologically proven gastric adenocarcinoma and curative intent gastrectomy with lymphadenectomy (D1 or D2) were included in this study. Patients who underwent conversion therapy, gastric remnant tumors, and emergency procedures were excluded.

Preoperative clinical data evaluated included hemoglobin and albumin levels (g/dL), neutrophil-to-lymphocyte ratio (NLR), American Society of Anesthesiologists classification (ASA), and Charlson-Deyo Comorbidity Index (CCI) without age and neoplasia in the score^12^. All patients underwent preoperative staging, consisting of computed tomography or magnetic resonance imaging of the abdomen and upper digestive endoscopy with biopsy; endoscopic ultrasonography was performed in selected cases. Tumor staging was based on TNM 8th edition^4^. Laboratory tests were also performed before surgery.

Preoperative CMT was indicated after a multidisciplinary team meeting (medical oncologist, surgeon, radiologist, and pathologist) in cases with clinical staging T2-T4 and/or positive LN^23^. The CMT regimen was chosen by the attending medical oncologist. Total or distal gastrectomy was performed according to the location and size of the tumor to achieve an R0 resection. The extent of LN dissection (D1 or D2) was defined by the attending surgeon responsible for the case. All procedures were performed according to the guidelines of the Japanese Gastric Cancer Association and the Brazilian Gastric Cancer Association by an extensively experienced surgical team^8,21^. The postoperative complication was graded according to the Clavien-Dindo classification, in which grades III to V were defined as the major postoperative complications (POC)^17^.

For analysis, patients were divided into two groups according to the initial surgical approach: surgery upfront (SURG) and preoperative chemotherapy (CMT), which included perioperative and neoadjuvant schemes. The outcomes evaluated included POC rate, disease-free survival (DFS), and overall survival (OS). Mortality at 30 and 90 days was also evaluated as short-term result. To reduce the effect of patient selection bias between the two treatment approaches, we performed the PSM analysis. Patients were followed up in the outpatient clinical appointments according to a standard protocol: every 3 months during the first year, every 6 months during the second and third years, and once a year thereafter. The study was approved by the Institutional Review Board of the hospital and registered online (www.plataformabrasil.saude.gov.br; CAEE: 59931922.0.0000.0068).

### Statistical analysis

Descriptive statistics were presented as frequencies for categorical variables, mean with standard deviation (±SD), or median with interquartile ranges (IQR) for continuous variables. Continuous and categorical variables were compared using the Student's *t-*test or Mann-Whitney U test and Pearson's χ² test, respectively.

PSM were calculated by bivariate logistic regression, including the following variables that might be considered potential confounders related to the selection and prognosis between groups: age (<65 vs =65 years), sex (female vs male), ASA (I/II vs III/IV), CCI (0 vs =1), hemoglobin levels (<11 vs =11g/dL)^13^, type of resection (distal vs total gastrectomy), tumor size (<5 vs =5cm), depth of tumor invasion (cT4 vs cT1-T3) and LN metastasis (cN0 vs cN+). The PSM was performed with a 1:1 distribution between groups with a caliper value of 0.01 (one-to-one nearest neighbor matching). The standardized difference (10% or 0.1) was used to compare the distribution of all paired covariates between the groups after PSM.

Survival rates were calculated using the Kaplan-Meier method, and differences between the curves were assessed using the logrank test. Prognostic factors associated with survival were estimated using Cox proportional hazards model. The variables with p<0.100 in univariate analysis were included in the multivariate model. DFS was calculated from surgery to recurrence or death from any cause, and OS was the duration between surgical resection to death. All statistical tests were two-sided and p-values<0.05 were considered significant. Statistical analyses were carried out using Statistical Package for Social Sciences (SPSS) software, version 20 (Chicago, IL).

## RESULTS

A total of 536 GC patients were eligible for inclusion. Among them, 424 (79.1%) underwent surgery upfront (SURG group) and 112 (20.9%) were referred for preoperative chemotherapy (CMT group). Among the CMT group, the most commonly prescribed regimen was 5- fluorouracil plus oxaliplatin in 36 patients (32.1%), followed by capecitabin plus platinum in 34 patients (30.3%), and cisplatin plus irinotecan in 26 patients (23.2%). The clinical and surgical characteristics of both groups before and after PSM are demonstrated in Table 1. The pathological characteristics and postoperative outcomes are in Table 2.

**Table 1:** Clinical and surgical characteristics of upfront surgery group and preoperative chemotherapy group, before and after propensity score matching.

		Before PSM		p-value	After PSM		p-value
		Upfront Surgery	Chemotherapy		Upfront Surgery	Chemotherapy	
		n=424 (%)	n=112 (%)		n=112 (%)	n=112 (%)	
Sex				0.059			0.375
	Female	174 (41)	35 (31.2)		29 (25.9)	35 (31.2)	
	Male	250 (59)	77 (68.8)		83 (74.1)	77 (68.8)	
Age (years)				0.037			0.344
	Mean (SD)	63.1 (12.5)	60.3 (12)		61.9 (19.9)	60.3 (12)	
BMI (Kg/cm²)				0.610			0.689
	Mean (SD)	24.1 (4.9)	23.9 (4.1)		24.1 (5.3)	23.9 (4.1)	
Hemoglobin (g/dL)				0.004			0.235
	Mean (SD)	12.1 (2.3)	11.5 (1.9)		11.8 (2.6)	11.5 (1.9)	
Albumin (g/dL)				0.769			0.229
	Mean (SD)	4.0 (1.6)	3.9 (0.5)		3.8 (0.7)	3.9 (0.5)	
NLR				0.022			0.029
	Mean (SD)	3.05 (3.06)	2.34 (2.07)		3.07 (2.87)	2.34 (2.07)	
ASA				0.487			0.298
	I/II	304 (71.7)	84 (75)		77 (68.8)	84 (75)	
	III/IV	120 (28.3)	28 (25)		35 (31.2)	28 (25)	
CCI				0.162			0.882
	0	277 (65.3)	81 (72.3)		80 (71.4)	81 (72.3)	
	=1	147 (34.7)	31 (27.7)		32 (28.6)	31 (27.7)	
cT				0.073			0.892
	cT1/T2/T3	281 (66.3)	64 (57.1)		65 (58)	64 (57.1)	
	cT4	143 (33.7)	48 (42.9)		47 (42)	48 (42.9)	
cN				<0.001			0.225
	cN0	138 (32.5)	17 (15.2)		11 (9.8)	17 (15.2)	
	cN+	286 (67.5)	95 (84.8)		101 (90.2)	95 (84.8)	
Type of resection				<0.001			0.564
	Subtotal	277 (65.3)	33 (29.5)		37 (29.4)	33 (29.5)	
	Total	147 (34.7)	79 (70.5)		75 (66.9)	79 (70.5)	
Surgical access				0.057			0.509
	Open	342 (80.7)	99 (88.4)		102 (91.1)	99 (88.4)	
	Minimally invasive	82 (19.3)	13 (11.6)		10 (8.9)	13 (11.6)	
Type of lymphadenectomy				0.060			0.538
	D1	77 (18.2)	12 (10.7)		15 (13.4)	12 (10.7)	
	D2	347 (81.8)	100 (89.3)		97 (86.6)	100 (89.3)	

PSM: propensity score matching; SD: standard deviation; BMI: body mass index; NLR: neutrophil-lymphocyte ratio; ASA: American Society of Anesthesiologists; CCI: Charlson-Deyo Comorbidity Index; cT: tumor metastasis at clinical stage; cN: node metastasis at clinical stage.

**Table 2:** Pathological and postoperative characteristics of upfront surgery group and preoperative chemotherapy group, before and after propensity score matching.

		Before PSM		p-value	After PSM		p-value
		Upfront Surgery	Chemotherapy		Upfront Surgery	Chemotherapy	
		n=424 (%)	n=112 (%)		n=112 (%)	n=112 (%)	
Tumor size (cm)				0.456			0.003
	Mean (SD)	5.4 (3)	5.2 (3.2)		6.6 (3.6)	5.2 (3.2)	
Lauren histological type				0.929			0.789
	Intestinal	214 (50.5)	56 (50)		58 (51.8)	56 (50)	
	Diffuse/mixed	210 (49.5)	56 (50)		54 (48.2)	56 (50)	
Grade of differentiation				0.918			0.496
	Well/moderate	184 (43.34)	48 (42.9)		43 (38.4)	48 (42.9)	
	Poorly	240 (56.6)	64 (57.1)		69 (61.6)	64 (57.1)	
Lymphatic invasion				0.002			<0.001
	Absent	163 (38.4)	61 (54.5)		34 (30.4)	61 (54.5)	
	Present	261 (61.6)	51 (45.5)		78 (69.6)	51 (45.5)	
Venous invasion				0.077			0.010
	Absent	237 (55.9)	71 (65.2)		54 (48.2)	71 (65.2)	
	Present	187 (44.1)	39 (34.8)		58 (51.8)	39 (34.8)	
Perineural invasion				0.012			0.003
	Absent	160 (37.7)	57 (50.9)		35 (31.2)	57 (50.9)	
	Present	264 (62.3)	55 (49.1)		77 (68.8)	55 (49.1)	
pT status*				0.024			0.001
	pT0/T1/T2	100 (23.6)	39 (34.8)		18 (16.1)	39 (34.8)	
	pT3	172 (40.6)	45 (402)		44 (39.3)	45 (40.2)	
	pT4	152 (35.8)	28 (25)		50 (44.6)	28 (25)	
Number of dissected lymph nodes				0.020			0.050
	Mean (SD)	42.1 (17.3)	47.2 (21.2)		42.0 (18.2)	47.2 (21.2)	
pN status*				0.016			<0.001
	pN0	110 (25.9)	42 (37.5)		13 (11.6)	42 (37.5)	
	pN+	314 (74.1)	70 (62.5)		99 (88.4)	70 (62.5)	
pTNM*				0.087			<0.001
	0/I/II	185 (43.6)	59 (52.7)		29 (25.9)	59 (52.7)	
	III/ IV	239 (56.4)	53 (47.3)		83 (74.1)	53 (47.3)	
Length of stay (days)				0.010			0.380
	Median (IQR)	11.5 (9–15)	11 (9–15)		11.5 (9–15)	11 (9–15)	
Postoperative complications				0.993			0.485
	None/Minors	256 (84)	94 (83.9)		90 (80.4)	94 (83.9)	
	Majors	68 (16)	18 (16.1)		22 (19.6)	18 (16.1)	
Mortality							
	30-day	16 (3.8)	5 (4.7)	0.589	7 (6.2)	5 (4.7)	0.620
	90-day	35 (8.4)	6 (5.7)	0.362	11 (9.9)	6 (5.7)	0.252
Adjuvant Chemotherapy				0.002			0.001
	No	173 (40.8)	64 (57.1)		39 (34.8)	64 (57.1)	
	Yes	251 (59.2)	48 (42.9)		73 (65.2)	48 (42.9)	

PSM: propensity score matching; SD: standard deviation; pT: postoperative p staging; pN: postoperative n staging; IQR: interquartile range.

* prefix “yp” for patients with preoperative

### Before propensity score matching

Patients of the SURG were older (p=0.037) and had a higher NLR (p=0.022) than the CMT. Lower hemoglobin level (p=0.004), higher rate of LN metastasis at clinical stage (cN) (p<0.001), and higher frequency of total gastrectomy (p<0.001) were associated with the CMT (Table 1).

Regarding pathological characteristics (Table 2), the absence of lymphatic (p=0.002) and perineural invasion (p=0.012), less advanced pT (p=0.024) and pN0 status (p=0.016) were associated with the CMT. There was no difference concerning the final TNM stage (p=0.087), frequency of POC (p=0.993), and mortality at 30 and 90 days between the groups. The frequency of postoperative chemotherapy was higher in the SURG (p=0.002)

### After propensity score matching analysis

**Figure 1:**
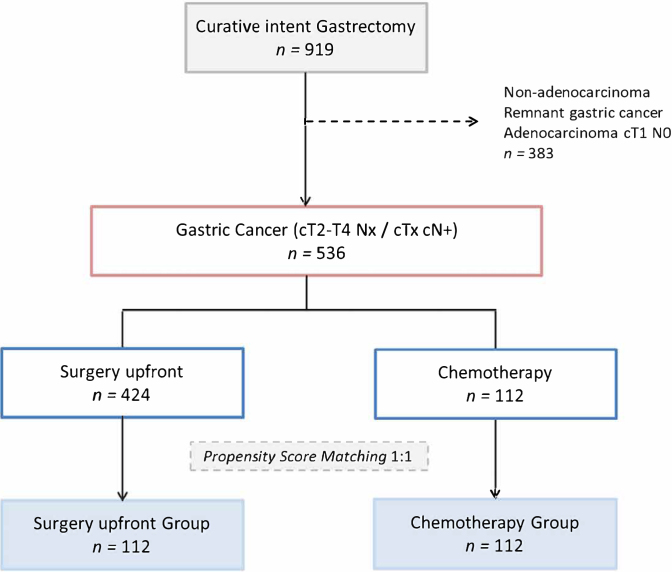
Study Flow chart.

**Figure 2:**
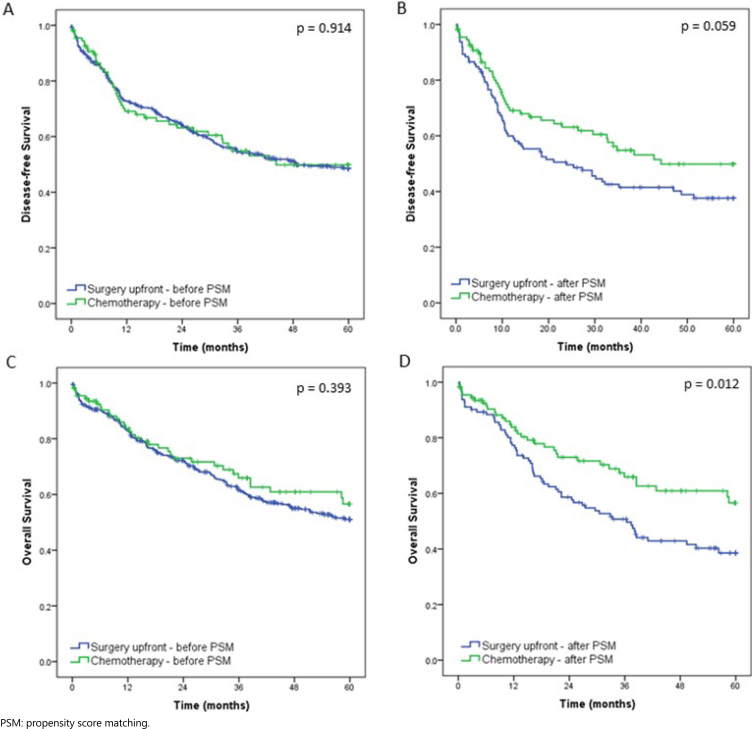
Disease-free survival and overall survival of patients who underwent preoperative chemotherapy and those treated with upfront surgery, before and after propensity score matching.

After PSM, using the 9 variables previously described, 112 patients were stratified for each group. A flow chart of the patient selection scheme is demonstrated in Figure 1. Histograms of propensity score distribution before and after PSM are presented in Figure 2.

As a result of the PSM, both groups were similar for all variables assigned in the score (Table 1). NLR was higher in the SURG (3.07 vs 2.34, p=0.029). Regarding pathological characteristics (Table 2), smaller macroscopic tumors (p=0.003), lower rates of lymphatic (p<0.001), venous (p=0.010), and perineural (p=0.003) invasions were related to the CMT (Table 3). The CMT group also showed less depth of tumor invasion (p<0.001), pN0 status (p<0.001), and less advanced TNM stage (p<0.001) compared to those in the SURG.

**Table 3:** Univariate and multivariate analyses for disease-free survival and overall survival, after propensity score matching.

Disease-free survival	Univariate		p-value	Multivariate*		p-value
	HR	95%CI		HR	95%CI	
Male (vs female)	1.01	0.67–1.53	0.944	–	–	–
Age =65 (vs <65 years)	1.06	0.72–1.56	0.761	–	–	–
CCI =1 (vs 0)	1.23	0.82–1.84	0.323	–	–	–
ASA III/IV (vs ASA I/II)	1.49	1.00–2.23	0.048	1.32	0.88–1.98	0.176
Total gastrectomy (vs distal)	1.17	0.78–1.75	0.452	–	–	–
Diffuse/mixed (vs others)	1.16	0.80–1.68	0.445	–	–	–
pT3/T4 (vs pT0/T1/T2)	1.68	1.04–2.74	0.035	1.18	0.71–1.97	0.524
pN+(vs pN0)	3.02	0.16–5.40	<0.001	2.66	1.43–4.95	0.002
Upfront surgery vs chemotherapy	1.44	0.98–2.11	0.061	1.09	0.73–1.62	0.682
**Overall survival**	**Univariate**		**p-value**	**Multivariate** *		**p-value**
**Variables**	**HR**	**95%CI**		**HR**	**95%CI**	
Male (vs female)	1.05	0.68–1.63	0.826	–	–	–
Age=65 (vs <65 years)	1.26	0.84–1.88	0.259	–	–	–
CCI=1 (vs 0)	1.41	0.92–2.15	0.114	–	–	–
ASA III/IV (vs ASA I/II)	1.85	1.22–2.80	0.004	1.60	1.05–2.44	0.027
Total gastrectomy (vs distal)	1.05	0.69–1.59	0.835	–	–	–
Diffuse/mixed (vs others)	1.07	0.72–1.59	0.739	–	–	–
pT3/T4 (vs pT0/T1/T2)	1.90	1.11–3.25	0.019	1.32	0.75–2.31	0.333
pN+(vs pN0)	3.00	1.60–5.63	0.001	2.34	1.19–4.59	0.013
Upfront surgery (vs chemotherapy)	1.69	1.11–2.55	0.013	1.25	0.81–1.93	0.304

HR: hazard ratio; CI: confidence interval; ASA: American Society of Anesthesiologists; CCI: Charlson-Deyo Comorbidity Index; pT: postoperative p staging; pN: postoperative n staging.

* Included in the multivariate model variables with p<0.100 in the univariate analysis.

The rate of postoperative complications and mortality remained similar between the groups even after PSM. Patients of the SURG performed more adjuvant chemotherapy (p=0.001).

### Survival analysis

**Figure 3:**
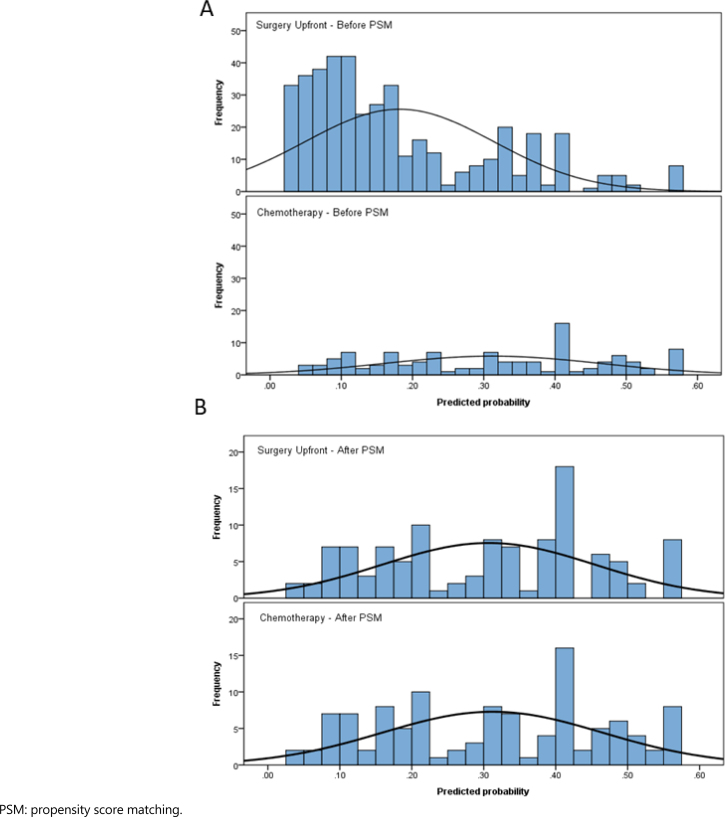
Histograms of propensity score distribution, before (A) and after (B) propensity score matching.

After a median follow-up of 33.5 months, 150 patients had disease recurrence and 215 died. The estimated DFS and OS rates of all cases were 48.7% and 52.4%, respectively. Survival curves before and after PSM are shown in the Figure 3.

There was no difference in DFS and OS between the two groups before matching (p=0.914 and p=0.393, respectively). After PSM, DFS was better in the CMT, although not reaching statistical significance (p=0.059). For OS, patients in the CMT had significantly longer survival compared to the SURG (p=0.012). The multivariate analysis after PSM demonstrated that the presence of LN metastasis was the only independent factor associated with poor DFS. Regarding OS, ASA III/IV category and pN+ status, they were factors significantly associated with worse survival in the multivariate model (Table 3).

## DISCUSSION

In this retrospective study, we evaluated the surgical and oncologic outcomes of patients with locally advanced GC who underwent upfront surgery compared to those who received preoperative CMT followed by surgery. Our objective was to evaluate whether preoperative CMT is better than upfront surgery for the treatment of advanced GC outside the context of clinical trials. Thus, we applied PSM analysis to minimize the impact of confounding variables on patient selection and prognosis between the two treatment groups. Hence, our findings demonstrated that patients who received perioperative CMT had better survival outcomes, with similar surgical results.

As expected, there was a trend to refer patients with more advanced diseases to perioperative CMT, which can be evidenced by the higher frequency of anemia, LN involvement, and total resections in this group. Interestingly, in our cohort, there was no difference between the groups regarding the presence of comorbidity and ASA category. An important aspect of our cohort is that patients who received perioperative CMT in the context of conversion therapy were excluded. As this group is considered beyond the limits of curative therapy and often requires combined resections, its inclusion could bias comparisons^26^. Even so, before the PSM there was an equivalent number of POC between groups, a fact that was maintained after the PSM. Thus, we can reaffirm the absence of a deleterious effect of CMT in POC in our population.

Analyzing the pathological findings, we observed that the CMT group had lower rates of lymphatic, venous, and perineural invasion. Similarly, we noticed that the pathological staging of the CMT group had a lower frequency of LN metastasis and a trend to lower pT4 stages, as also observed in our previous study^16^. As previously mentioned, one of the primary goals of CMT is tumor downstaging. Since we found patients with a less advanced stage in the CMT group compared to the SURG group – despite both being similar in clinical staging – our findings confirm the role of preoperative CMT in promoting tumor downstaging. This characteristic has been greatly explored in several studies and trials, where the response to CMT would be the main factor responsible for the survival advantage in those patients^1,2,9,11^. However, it is still debated whether the degree of tumor regression, the final ypTNM stage, or only the LN status would have greater importance in the prognosis^22,24,32,34^.

It was found that the SURG group received more adjuvant CMT. This difference was expected, once some preoperative regimens consisted of only neoadjuvant CMT. However, this difference may have been accentuated by the fact that the medical oncologist felt more comfortable not prescribing the second part of the scheme since a certain amount of CMT had already been administered. This fact is particularly relevant in more fragile patients and in those who had POC, which could prevent them to return to the intended oncological treatment after surgery^25^. Further, another aspect of such difference refers to the fact that patients in the SURG group had a more advanced pathological stage and, therefore, were more often referred for adjuvant CMT.

As for the OS rate, we noted that, after PSM, the CMT group had improvement in OS and a trend to better DFS compared to the SURG. These findings in our local setting corroborate the results obtained in previous RCTs. Even with clinical and social characteristics peculiar to our country, which include advanced tumors in malnourished patients with considerable social risk, perioperative CMT had a positive impact on survival^14^. However, it is important to address as a limitation of our study, the fact that we only evaluated patients of the CMT group who were able to reach the surgery. Consequently, it did not allow us to identify the percentage of patients who were unable to complete preoperative CMT, or those that had disease progression during its administration.

The inclusion of GEJ tumors and the predominance of proximal tumors in some studies are frequently highlighted as a difference to our daily practice^15,29,33^. If, on the one hand, the greater occurrence of distal tumors reduces the higher surgical risks of total gastrectomy, proximal and GEJ tumors present more exuberant clinical and pathological responses to CMT than distal GC^18,31^. This ratio of subtotal/total resections is exactly the opposite of what was previously verified in our historical cohort^27^. This reflects the greater trend to indicate proximal tumors to CMT based on the already available literature.

Although the present study shows the effectiveness of preoperative CMT in our service, the inclusion of patients from a single center may limit the generalization of the results. In addition, the long inclusion period and different CMT regimens may also contribute to the variation in observed results. Unfortunately, the FLOT scheme, which is currently the most recommended, was not used in our patients^3,23^. Finally, our cohort of patients referred for preoperative CMT is still relatively small compared to patients treated with upfront surgery, which makes it impossible to assess the subgroups that would benefit more or less from the multimodal treatment approach.

Despite these limitations, we were able to demonstrate that preoperative CMT represents an effective strategy for the treatment of advanced GC in our service, offering distinct advantages over upfront surgery. To date, a multimodal treatment approach, incorporating both CMT and surgery, can achieve the best possible outcomes in advanced GC, and the next steps include the development of new schemes that can further increase survival, such as the current incorporation of immunotherapies in recent RCTs^6,20^. In this setting, the great challenge will be to identify those patients who will benefit from a specific regimen and tailor treatment to the individual patient's disease characteristics and overall health status. Thus, maintaining the analysis of treatment effects in different regions and cohorts of patients is still necessary to verify the most appropriate treatment approach.

## CONCLUSIONS

Preoperative CMT provided better survival outcomes for patients with GC. There was no difference in the POC rate and mortality before and after PSM between both approaches, but the CMT group had a less advanced stage compared to the patients treated with upfront surgery.
